# The Achilles Heel of Technology: How Does Technostress Affect University Students’ Wellbeing and Technology-Enhanced Learning

**DOI:** 10.3390/ijerph182312322

**Published:** 2021-11-24

**Authors:** Xinghua Wang, Zhenyu Li, Zhangdong Ouyang, Yanping Xu

**Affiliations:** 1 Normal College, Qingdao University, Qingdao 266071, China; wangxinghua379@gmail.com (X.W.); xuyanping97@126.com (Y.X.); 2 School of Economics, Qingdao University, Qingdao 266071, China; 3 School of Mathematics and Computational Sciences, Hunan First Normal University, Changsha 410205, China; oymath@163.com

**Keywords:** stressor-strain-outcome model, technostress creators, technostress inhibitors, wellbeing, technology-enhanced learning

## Abstract

This study investigated the effect of technostress on university students’ wellbeing and technology-enhanced learning (TEL) through the stressor-strain-outcome model. Interviews were first used to contextualize and inform the development of the survey instrument. Then, survey data from 796 participants were collected and analyzed using partial least squares structural equation modeling. The findings indicate that technostress creators, including techno-complexity, techno-insecurity, and techno-uncertainty, were significantly associated with students’ burnout in TEL, which in turn was negatively associated with their self-regulation, learning agency, and persistence in TEL. Group comparison analyses based on gender, academic disciplines, and willingness to join TEL show that the negative associations between burnout and self-regulation, learning agency, and persistence in TEL were significantly stronger for male students than female students. Similar findings were also found for students joining TEL willingly and unwillingly, with the latter being more strongly affected by burnout. In addition, the positive association between techno-complexity and burnout was greater for students from social sciences than those from engineering and natural sciences. The findings of this study can inform future implementation decisions of TEL in higher education and strategies to preserve university students’ wellbeing.

## 1. Introduction

Universities around the world have been ardently innovating learning and teaching through digital technology, such as intelligent tutoring systems, virtual learning environments, mobile computing devices, and artificial intelligence-powered applications [1,2,3]. The various forms of learning that are facilitated by digital technology are broadly called technology-enhanced learning (TEL) [4]. The reasons behind institutional commitments in TEL are related to the potential benefits of TEL, for instance, increasing student engagement and achievement, enabling more flexibility in learning and teaching, and enlarging access to quality resources [5,6].

However, TEL often involves changes in established learning and teaching practice and requires stronger self-regulation and time management abilities, thus incurring changed expectations of university students [2]. Eventually, those who are not used to TEL may experience technostress, which, in the context of this study, is defined as a maladaptation problem caused by individuals’ incapability to cope with the demands of technology and changing requirements associated with the use of technology in their work in a healthy manner [1,7,8]. As the potentials of digital technology are determined by the way in which it is used, it makes little sense to discuss technology without the pedagogy revolving around it [5]. Therefore, technostress in the field of education is related to not only technical issues but pedagogical issues, in particular.

Technostress can lead to a variety of negative consequences to individuals’ psychological and physiological health, for instance, frustration, anxiety, and fatigue [9,10]. It can further adversely affect their work, such as causing concentration problems, biasing their judgements of digital technology, and decreasing work performance [11,12]. Students with high technostress may reduce their involvement in TEL and even quit TEL entirely [2,13]. As such, the issue of technostress is subject to due attention from educational practitioners in higher education.

Previous research on technostress mainly focused on employees in the workplace [14,15] and a limited amount of research attention has also been given to the teacher population, for instance, secondary teachers [9,11] and academics in higher education [10]. However, there has been a dearth of studies investigating technostress experienced by university students who are often the main users of digital technology in higher education, which has been continually shaped and reshaped by digital technology [4,13].

Therefore, to preserve university students’ wellbeing and refine future implementation of TEL in higher education, this study aimed to investigate how technostress affects students’ learning in TEL. The stressor-strain-outcome (SSO) model was used for this purpose as it provides a parsimonious theoretical framework to explain how the maladaptation to TEL predisposes university students to strain and consequently causes outcomes for their learning.

In addition, as prior studies [16,17] diverge on the mediating effect of gender on technostress and its impact on individuals’ life and work, this study will continue the exploration of this topic to further examine the relationship between demographics and technostress. As the participants in this study mainly came from two disciplines (social sciences: Psychology and educational science; engineering and natural sciences: Material science, electric engineering, and computer science), it will be helpful for future improvement decisions of TEL by examining how students from different disciplines may differ in their experience of technostress. Finally, based on our knowledge of the research context, we found that some TEL courses were compulsory while others were not. As individuals tend to behave differently based on their willingness to join activities [18], examining how students’ willingness to join TEL may affect their technostress will inform future implementation of TEL in different courses. Therefore, this study aims to address the following research questions:

(1) How does technostress affect students’ learning in TEL via burnout?
(2) How do factors such as gender, disciplines, and willingness to join TEL moderate the relationships among technostress, burnout, and students’ learning in TEL?

The remainder of this paper is organized in the following ways. Section 2 presents the theoretical framework in which prior studies on technostress, the SSO model, and the proposed research model of this study are given. Section 3 focuses on the methodology where the participants, the research context, the instrument, and the technique for data analysis are presented. Afterwards, Section 4 reports the research findings, which are discussed in Section 5 along with the contributions, implications, and limitations. This study is concluded in Section 6.

## 2. Theoretical Framework

### 2.1. Literature on Technostress

Although digital technology has been used prevalently in every aspect of our society, technostress has been an understudied topic [19]. Current research on this issue has been mainly conducted in the sectors of industry and government [14,20]. It has been raised by people using various forms of technology, such as mobile devices [21], corporate management technology [12], digital textbooks [13], and collaborative tools [10].

Despite the increasing number of studies on technostress experienced by different user populations, there has been very limited research on it in the field of education, especially among the student population [8,13]. It may be because of the widely-held stereotype of the young generation of learners, who are often perceived to be tech-savvy [10]. However, this perspective may be applicable when young students are using technology for entertainment or personal interest. They may still experience technostress when the technology is being used intensively for learning purposes in school settings [22]. Considering the increasing investment in digital technology and the widespread adoption of TEL to transform conventional education in higher education institutions [2,4,23], technostress is likely to be pervasive among university students.

According to previous technostress studies, including Hwang and Cha [15], Marchiori et al. [20], and Ragu-Nathan et al. [17] across different fields, five typical forms of technostress creators, which are not exhaustive, have often been raised: Techno-overload, techno-invasion, techno-complexity, techno-insecurity, and techno-uncertainty. In line with these studies while considering the context of the current research, the definitions of the five technostress creators are given as follows.

Techno-overload is related to the scenario where university students are driven to learn faster and longer because of increased learning demands in TEL. Techno-invasion refers to the situation in which the integration of digital technology in learning settings pushes university students to be connected constantly and reached anytime such that their personal lives are invaded. Techno-complexity is related to the situation where TEL increases the difficulty of students’ work and there is a long learning curve for students to adapt to it. Techno-insecurity describes the scenario where students feel insecure about the prospect of their established learning habits being disrupted by TEL and they have to frequently learn and relearn skills to cope with new requirements of TEL. Finally, techno-uncertainty refers to the situation where TEL disrupts students’ study plans and creates ambiguous learning expectations. In the present study, we will examine how the five technostress creators impact students’ learning through the SSO model.

### 2.2. Stressor-Strain-Outcome (SSO) Model

The SSO model was originally developed by Koeske and Koeske [24] to illustrate how stressors affect individuals’ work and life. According to this model, stressors are associated with outcomes via the mediating role of strain. In the current study, the main reasons behind adopting the SSO model over other models such as the transactional model of stress (refer to Lazarus and Folkman [25]) are as follows. The SSO model has been widely used to investigate stress in different fields such as industries and the field of education [26,27]. It provides a simple yet effective framework to explain the dynamic of technostress and its impact on individuals’ health and work [14,28,29]. More importantly, the SSO model neatly fits the objective of this study, which is to examine how technostress creators (i.e., stressors) may affect students’ learning.

In the SSO model, stressors refer to stimuli of stress that are perceived by individuals as troublesome. In the context of the present study, students’ maladaptation to TEL is considered stressors, which are also called technostress creators. Strain emerges from stressors and refers to the disruptive influence on individuals’ emotions and psychology. It is embodied in the original SSO model by the construct of burnout, which refers to the state of physical and psychological exhaustion and fatigue and mediates the impact of stressors on individuals’ psychological or behavioral outcomes related to work [24,26,30]. Finally, outcomes arise from strain and refer to undesirable psychological or behavioral consequences for individuals’ life or work.

In line with the SSO framework and prior research on technostress [13,29], we posit that technostress creators (stressors) induce burnout (strain), which, in turn, causes negative outcomes for students’ learning performance (i.e., self-regulation, learning agency, and persistence in TEL). Specifically, the following hypotheses are proposed:

**Hypothesis** **1** **(H1).** *Techno-overload is positively associated with burnout in TEL.*

**Hypothesis** **2** **(H2).** *Techno-invasion is positively associated with burnout in TEL.*

**Hypothesis** **3** **(H3).** *Techno-complexity is positively associated with burnout in TEL.*

**Hypothesis** **4** **(H4).** *Techno-insecurity is positively associated with burnout in TEL.*

**Hypothesis** **5** **(H5).** *Techno-uncertainty is positively associated with burnout in TEL.*

**Hypothesis** **6** **(H6).** *Burnout is negatively associated with self-regulation in TEL.*

**Hypothesis** **7** **(H7).** *Burnout is negatively associated with learning agency in TEL.*

**Hypothesis** **8** **(H8).** *Burnout is negatively associated with persistence in TEL.*

**Figure 1:**
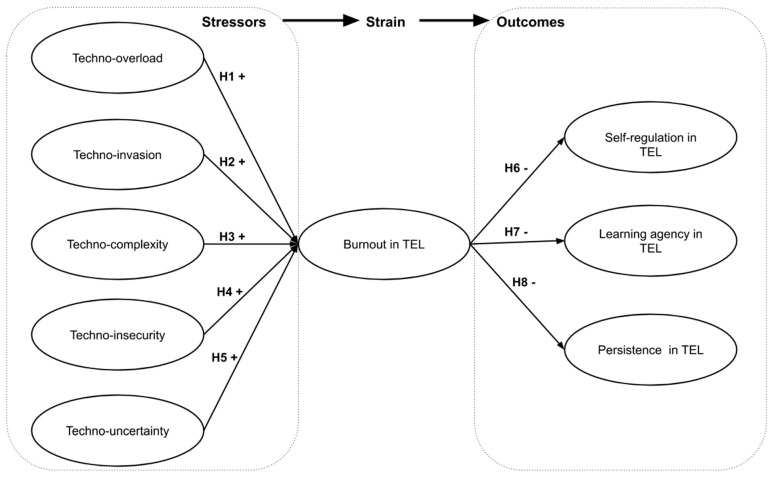
Hypothesized stressor-strain-outcome (SSO) model of technostress. Note. “+” suggests positive relationships; “−” suggests negative relationships.

The research model is visualized in Figure 1.

The constructs of self-regulation, learning agency, and persistence are used to indicate university students’ performance in the present study due to the following reasons. Self-regulation is concerned with the internal locus of control over and accountability of one’s learning activities and is of vital importance to the success in TEL where students often need to decide what, when, and how to study [23]. Effort regulation, time management, and attentional focus are a few examples of self-regulatory strategies university students often employ to attain desired goals [31]. Learning agency is related to the ability to take up learning responsibilities and opportunities and control one’s own time and pace in a learning setting [32,33]. More meaningful learning occurs when students take up higher levels of learning agency [34]. Nevertheless, students experiencing technostress are likely to doubt the ownership of learning in TEL and decrease their agency in active participation in TEL. In addition, persistence in TEL is also essential for the success of TEL [35]. Without consistent investment of efforts and time, university students are not likely to benefit from TEL.

As Maslach et al. [36] pointed out, burnout is not simply individuals’ experience of physical and psychological exhaustion but also incurs actions to distance themselves cognitively and emotionally from the work that causes the negative experience for them. Students experiencing burnout tend to reduce their negative feelings by decreasing their learning commitment and adopting withdrawal coping strategies [27]. Therefore, we hypothesize that burnout is negatively associated with university students’ self-regulation, learning agency, and persistence in TEL.

## 3. Methodology

### 3.1. Participants and Research Context

The data were collected from students of three large public universities (the focal university hereafter) in northern China. The universities were similar in size, student demographics, and rankings in academic strength. As female students far outnumbered males in the focal universities, we approached the administrative offices in the universities for help in obtaining participants with a more balanced gender distribution. Among the 950 participants we approached using convenience sampling, valid responses from 796 of them were attained with their informed consent, among whom there were 381 males and 415 females with the age ranging from 18 to 23 years. There were 334 of them studying social sciences and 462 studying engineering and natural sciences. Most of the participants (568 students) indicated that they joined TEL willingly while 228 participants indicated that they joined TEL against their willingness.

The focal universities had been supportive of the implementation of TEL by making favorable policies and setting up special funds for it. For instance, faculty who intended to make small private online courses would be fully subsidized. Those who were experimenting with a flipped classroom would get more credit in yearly performance appraisals. Some TEL were implemented from the institutional level, for example, by introducing massive open online courses (MOOCs) to their curricula and assigning credit to them. Others were carried out by different departments and faculty based on their actual needs. For instance, vendors specializing in application development collaborated with faculty to transform their traditional courses to flipped classroom. Mobile learning was also quite popular thanks to the ubiquity of smartphones. Nevertheless, despite the benign intentions on the part of the universities to use digital technology to increase students’ engagement and achievement, there are often mismatches between universities’ expectations and students’ reactions [2].

### 3.2. Focus Group Interviews

To contextualize the hypotheses and adapt the survey items in this study as well as to flesh out the quantitative findings, focus group interviews were conducted as they offered an efficient way of gaining in-depth insights into students’ positive and maladaptive experiences of TEL [37]. Forty-one students were selected using purposive sampling, with 10 students from electronic engineering, physics, and psychology each and 11 students from educational sciences. Each focus group interview was conducted with 5 to 6 students and lasted approximately 30 min. To counteract potential disadvantages of focus group interviews, such as biased results due to dominant group members, responses were cross-checked across individuals within a group and between groups. Focus group interviews were semi-structured and were organized around questions such as: How is your experience with TEL? What are your perceptions of the five technostress creators, if there are any? The interviews were audio-recorded and transcribed and were analyzed with Excel spreadsheets.

The interview transcripts were analyzed by relating them back and forth to the hypotheses and survey items with the objective of contextualizing and adapting them to the current study. In doing so, we sought to underline how technostress may arise when the students from different disciplines engage in TEL in which the use of digital technology and changed learning requirements challenge students’ established learning practice and compel them to spend more time and effort to adapt to the challenges, thereby imposing increasing levels of stresses on the students. Most students indeed supported the implementation of TEL in their universities as they perceived that TEL benefited them substantially. However, many of them also pointed out the problems in TEL, which can be conceptualized in terms of technostress.

For instance, TEL increased the complexity of students’ work and slowed down their learning progress. Echoing previous studies such as Selwyn [2], some students we interviewed complained that the learning resources in TEL did not have consistent designs. Some courses in the learning management system (LMS) were designed with such a bewildering labyrinth of headings or were filled with so many materials that it made it very difficult for students to look for information relevant to their work. There were also students complaining about the poorly designed LMS itself, where the catalogues of functions were very convoluted for the students to navigate through. As a result, they had to spend more time on performing learning tasks online than completing equivalent tasks offline. All these issues tended to increase the difficulty levels of students’ work in TEL, further adding to their stresses. Consequently, their learning agency, persistence in TEL, and self-regulation might be compromised [8,38].

In addition, to flesh out the quantitative results, thematic analysis was also used. The interview transcripts were coded using the quantitative findings as a starting list of codes by following the analysis procedures suggested by Creswell [39]. However, it was possible that not all quantitative findings would be related to the interview transcripts as the focus group interviews were conducted before statistical modeling. Representative quotations from the focus group interviews were selected to highlight and elaborate on some key findings from statistical modeling. Throughout the process of qualitative analyses, researchers reiteratively analyzed and discussed the data until all disagreements were settled.

### 3.3. Instrumentation

The survey instrument consists of three sections, including stressors (techno-overload (four items), techno-invasion (four items), techno-complexity (five items), techno-insecurity (four items), and techno-uncertainty (five items)), strain (burnout (six items)), and negative outcomes for learning performance (self-regulation (six items), learning agency (four items), and persistence in TEL (five items)). The items of the constructs were rated on a five-point Likert scale where 0 = strongly disagree and 4 = strongly agree. The survey was developed through several iterative refinements before it was administered to all participants. The original survey adapted from previous studies was first reviewed by three experts in the field of TEL, who critiqued the survey items for validity. Then the improved version of the survey was trailed by 10 student participants to test their understanding of the items. Those causing possible confusions were further refined. Considering that the survey was in English, we used a back-translation procedure to minimize differences between the English and Chinese versions.

Specifically, the five technostress creators were adapted from Ragu-Nathan et al. [17] and Fuglseth and Sørebø [29]. The Cronbach’s alpha values of the five technostress creators were reported to range from 0.77 to 0.87. The items for each technostress creator were essentially negatively worded. For instance, to indicate techno-uncertainty, we have items such as “I am uncertain about the usefulness of technology-enhanced learning”; to indicate techno-invasion, we have items such as “I have less free time due to the implementation of technology-enhanced learning”. Burnout was adapted from Kristensen et al. [30], including items such as “I feel overwhelmed due to technology-enhanced learning”. The Cronbach’s alpha value was reported to be 0.87. The construct of self-regulation was adapted from Barnard et al. [23]. Sample items included “I set clear goals in technology-enhanced learning”. The reported Cronbach’s alpha value was 0.78. Learning agency was adapted from Kearney et al. [32], with a reported Cronbach’s alpha value of approximately 0.78. Sample items included “I am active in participating in technology-enhanced learning”. Finally, the items measuring persistence in TEL were adapted from Wu and Chen [35], with a reported Cronbach’s alpha value of 0.94. Sample items contained “I persist in completing technology-enhanced courses that I have registered for”. As the items of the five technostress creators and burnout showed strong negative connotations while the remaining three constructs were positively worded, to align the scoring of all items, the item scoring of self-regulation, learning agency, and persistence in TEL were reversed [40].

### 3.4. Analysis of Survey Data

The survey data of this study were analyzed using the partial least squares structural equation modeling (PLS-SEM), which is a variance-based SEM [41]. It is prediction-oriented and exploratory in essence with the aim of maximizing the variance explained for dependent variables. Compared with the covariance-based SEM, which is confirmatory and theory-oriented, PLS-SEM does not require big sample sizes and multivariate normality assumption, and avoids the issues of factor indeterminacy and non-convergence [42]. As the present study sought to explore how technostress creators could predict students’ TEL via burnout, PLS-SEM fitted the purpose of this study. According to Hair et al. [43], a two-step analytical procedure was adopted, with the measurement model being examined first followed by the structural model. Subsequently, group comparisons were conducted to explore how the relationships among technostress, burnout, and students’ learning in TEL were affected by the students of different genders, disciplines, and willingness to join TEL.

## 4. Results

### 4.1. Validating the Measures

To establish the quality of the measures in the current study, an initial round of reliability checks and confirmatory factor analysis (CFA) was performed. Following that, common method bias was examined. In the end, the proposed technostress model was tested through PLS-SEM using the PLS-SEM package [44].

In the initial round of reliability analysis, the Cronbach’s alpha values of all the variables were as follows: Techno-overload (0.93), techno-invasion (0.91), techno-complexity (0.93), techno-insecurity (0.85), techno-uncertainty (0.94), burnout (0.95), self-regulation (0.93), learning agency (0.93), and persistence (0.93), all exceeding 0.70 and hence indicating high reliability. According to Cangur and Ercan [45] and Hooper et al. [46], the CFA was assessed based on the following criteria: (a) 2.0 ≤ the normed chi-square (χ^2^/df) ≤ 5.0; (b) the comparative fit index (CFI) ≥ 0.90; (c) normed fit index (NFI) ≥ 0.90; (d) standardized root mean square residual (SRMR) < 0.05, and (e) root mean square error of approximation (RMSEA) ≤ 0.08. In this study, the outcomes from the confirmatory factor analysis suggest satisfactory model fit, with χ^2^ = 3580.94, *p* < 0.001, χ^2^/df = 3.46, CFI = 0.94, NFI = 0.91, SRMR = 0.03, and RMSEA = 0.06.

Harman’s single-factor analysis [47] was used to detect potential common method variance, which could negatively affect the finding of the study. When all indicators were loaded onto one factor, the total variance for the single factor was 18.82%, which was lower than 50%, implying a low possibility of committing common method bias. Subsequently, PLS-SEM was used to compute the proposed technostress model. Two items in technostress-complexity and one item in technostress-insecurity were removed due to low discriminant validity. Eventually, the measurement model demonstrated strong reliability and validity (see Table 1 and Table 2).

### 4.2. Structural Model

The path coefficients in the technostress model, the endogenous factors’ explanatory power (*R*^2^), and the global goodness-of-fit (GoF) value were used to assess the structural model. With regard to the path coefficients, the bootstrapping method was applied to further validate them because PLS-SEM is not based on distributional assumption and thus parametric techniques are not appropriate [44,48].

**Figure 2:**
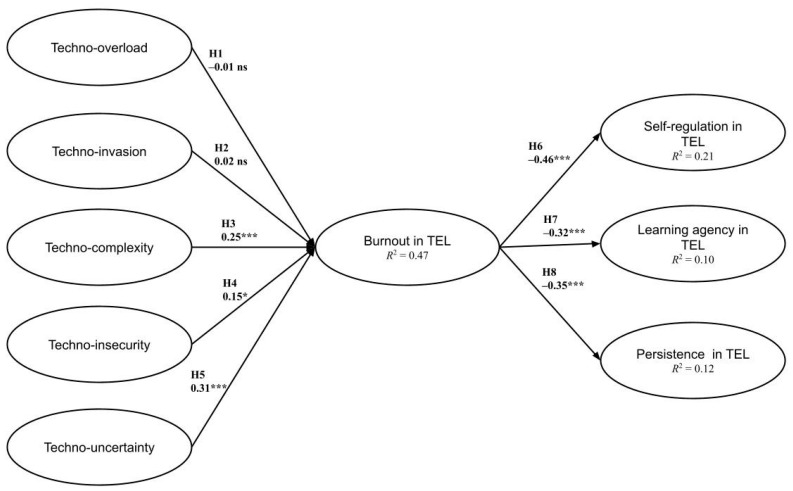
Validated SSO model of technostress. Note. * *p* < 0.05; *** *p* < 0.001; ns = non-significant.

Table 3 demonstrates the bootstrapping validation outcomes, which are visualized in Figure 2. The outcomes are generally in line with the SSO model and support the association between technostress and learners’ negative responses in TEL. Specifically, techno-complexity, techno-insecurity, and techno-uncertainty were positively associated with burnout while techno-overload and techno-invasion were not. Therefore, Hypotheses 3–5 were substantiated while Hypotheses 1 and 2 were not. In addition, as hypothesized, the negative associations between burnout and students’ self-regulation, learning agency, and persistence in TEL were significant, thus, substantiating Hypotheses 6–8.

The *R*^2^ values of the endogenous variables are often used as important criteria to determine the structural models’ quality because of the aim of PLS-SEM, which is to maximize the variance accounted for in endogenous variables [49]. In this study, the *R*^2^ values of burnout, self-regulation, learning agency, and persistence in TEL are 0.47, 0.21, 0.10, and 0.12 (see Figure 2), respectively, indicating medium-to-large effect sizes [50].

In addition, according to the global GoF proposed by Tenenhaus et al. [51], values of 0.10, 0.25, and 0.36 represent a small, medium, and large fit, respectively. The technostress model in this study generated a GoF value of 0.42, implying a good fit of the technostress model with the data.

### 4.3. Group Comparisons in Terms of Genders, Academic Disciplines, and Willingness to Join TELL

To explore whether gender, academic disciplines, and willingness to join TEL make a difference in the associations between technostress creators and students’ outcomes in TEL, multiple group comparisons were performed by comparing differences at the path coefficients of the technostress model [44,48].

The whole dataset was split into different sub-datasets according to gender, academic disciplines, and willingness to join TEL. Then the bootstrap *t*-test method was conducted to examine potential differences in terms of the path coefficients among different variables between sub-datasets.

#### 4.3.1. Gender Differences in the Technostress Model

Table 4 indicates that there are significant differences between male and female students in terms of (a) the path coefficient of burnout on self-regulation in TEL, *t* (794) = 2.59, *p* < 0.01, (b) the path coefficient of burnout on learning agency in TEL, *t* (794) = 2.45, *p* < 0.01, and (c) the path coefficient of burnout on persistence in TEL, *t* (794) = 2.50, *p* < 0.01. The differences imply that burnout may cause a more significant negative influence on male students’ self-regulation, learning agency, and persistence in TEL than females.

#### 4.3.2. Differences Based on Students’ Academic Disciplines

As shown in Table 5, there is a significant difference between students from social sciences and engineering and natural sciences in respect to the path coefficient of techno-complexity on burnout in TEL, *t* (794) = 1.94, *p* < 0.05, implying that techno-complexity may cause more burnout for students from social sciences than those from engineering and natural sciences.

#### 4.3.3. Differences Based on Willingness to Join TEL

Table 6 show that there are significant differences between students who joined TEL willingly and unwillingly in terms of the path coefficients of burnout on self-regulation (*t* (794) = 2.94, *p* < 0.01), learning agency (*t* (794) = 3.75, *p* < 0.001), and persistence in TEL (*t* (794) = 4.12, *p* < 0.001). This suggests that burnout may lead to a stronger negative influence on students who participate in TEL unwillingly than those who did so willingly.

### 4.4. Relating Qualitative Findings to Statistical Modelling

By relating the qualitative findings to statistical modeling, we hoped to gain an in-depth understanding of the emergence of technostress in TEL and its impacts on learners’ wellbeing and performance. Overall, the qualitative analyses of the focus group interviews largely corroborated the statistical modelling, excerpt for the group comparison outcomes, which were not easily discernible from interviews.

As many courses were using mobile LMSs to support blended learning, techno-overload and techno-invasion had become quite common in students’ daily lives. Nonetheless, the interviewed participants did not seem to feel frustrated towards these issues. One participant, for instance, stated:

I have five courses using YuKeTang (a mobile LMS). It seems to push notifications to me after whatever the instructors have done on it, for instance, uploading new learning materials, making course announcements, and even making a slight adjustment to course materials. As a result, I normally receive dozens of notifications every day, including weekends. Some notifications require me to respond immediately or within a short time frame, regardless of whether I am taking a different course or having meals at that time. At first, I find them very annoying. But now, I have already gotten used to them. (Student 2 with a Psychology major).

This phenomenon was usual for the participants who were taking several blended learning courses and largely bore out the statistical findings of insignificant negative effects of techno-overload and techno-invasion on students’ academic performance and wellbeing.

Techno-insecurity was mainly manifested in scenarios where students were forced to change their learning habits and felt stressed and insecure to adapt to new ones. As indicated by a student majoring in educational sciences but taking a math course, “math is already very difficult for me. But our instructor insists on using YuKeTang for assignments. Typing the formulas in computers is killing me…why not stay with paper-based assignments where I can freely scribble…” Even though YuKeTang facilitated quick feedback and flexibility in learning, the student did not seem to appreciate the advantages. Techno-complexity has been described in Section 3.2 when contextualizing the hypotheses of this study. As the hypothesized positive relationship between techno-complexity and burnout was substantiated, we will not elaborate on this hypothesis here to avoid redundancy.

Techno-uncertainty emerged when students were uncertain about what exactly TEL expected from them. The feelings of uncertainty toward TEL might lead students to self-doubt and feel strained. The experience of techno-uncertainty and consequent burnout seemed to be particularly frequent among the students taking recorded online courses. The focus group interviews offered a deeper understanding of this phenomenon, as evinced by a student majoring in electronic engineering:

I am in the top five percent of my class. However, I often feel at a loss when it comes to online courses as I do not perceive clear course instructions. The learning objectives in the (online) courses are blurry and confusing. (Student 4 with an electronic engineering major).

Another student experienced similar challenges:

In face-to-face courses, our instructors usually reiterate their course requirements now and then and guide us on how to develop proper learning strategies. But online instructors only focus on presenting content knowledge… I often end up feeling disoriented and demotivated. (Student 1 majoring in physics).

An immediate consequence of techno-uncertainty was decreased engagement, which can be conceived as reduced self-regulation, agency, and persistence in TEL. As opined by a student majoring in psychology, “we simply want to pass the courses rather than obtaining the highest scores…”

## 5. Discussion

This study aimed to investigate the issue of technostress among students in higher education through the lens of the SSO model. Most hypotheses were substantiated and thus largely support the effectiveness of the SSO model in explaining how technostress creators may lead to negative impacts on students’ outcomes in TEL. The results related to six out of the eight hypotheses (Hypotheses 3–8), provided further evidence-based support to the finding of prior studies about the consequences of technostress for individuals’ work and life [10,13,37].

The result that techno-overload was not significantly related to burnout (Hypothesis 1) seems to be unreasonable at first sight. Nevertheless, to a certain extent, it seems to be resonant with Hung et al. [21], who found that techno-overload actually improved individuals’ performance at work. This could be because even though TEL may require more investment of time and effort than conventional pedagogy, it provides convenience and quality resources for students, thereby likely counteracting the strain caused by overload. The result that techno-invasion was not greatly associated with burnout (Hypothesis 2) may be because that the participants in this study were full-time students mostly living inside the campuses, which also provide residential education. For them, campus life is often a mix of formal education in classrooms and informal education in private spaces. Therefore, they may have been used to the blurred boundary between TEL and their private lives.

Nevertheless, the findings in this study are quite different from Qi [22], which found that the academic use of mobile devices did not cause technostress. The reason may be that in Qi’s study, mobile devices were only used as media to deliver learning and teaching resources. There was almost no sign of pedagogical interventions related to mobile devices. The structure of learning and teaching was not reshaped by the integration of mobile devices. In other words, mobile devices were not used in a way that challenged students’ established learning habits. However, TEL, such as blended learning and accredited MOOCs in the focal universities, may have already challenged or changed students’ learning practice, consequently causing varying levels of technostress for students.

Burnout had stronger negative relationships with self-regulation, learning agency, and persistence in TEL for male students than females. This finding corroborates Ragu-Nathan et al. [17] who found that males experienced greater technostress and suffered from more consequences of technostress than females. This may be related to the characteristics of Chinese university students, among whom female students often demonstrate more positive attitudes toward learning than males and are more adaptive to changes caused by the integration of technology in learning [52]. Consequently, burnout caused by technostress in TEL may have relatively lower negative effects on females than males.

Burnout had greater negative associations with self-regulation, learning agency, and persistence in TEL for students joining TEL unwillingly than those who did so willingly. This result is largely in line with previous studies such as Sumiyana and Sriwidharmanely [18], which found that activities that are against students’ willingness may cause more adverse effects for students than those aligned with their willingness. When technology is used for serious purposes and compulsory for students, the entertaining side of technology may lose its luster and the coercive characteristic of TEL may disrupt students’ agency and internal control over their behaviors, eventually distancing students from persisted engagement in TEL.

Techno-complexity, among the five technostress creators, was more strongly associated with burnout for students in social sciences than those in engineering and natural sciences. It is quite challenging to give this finding a convincing explanation. Different academic disciplines have different training paradigms. Students in each discipline tend to develop their own patterns of reasoning and understanding and consequently, perceptions of their learning environments, which are closely linked to the characteristics and structures of their knowledge domains [53]. Students with a science and engineering background tend to express significantly stronger preferences for a logical learning style, which emphasizes logic and reasoning, than those with a social science background [54]. Therefore, they may be more proficient in dealing with complexity in learning environments than those in social sciences. As a result, the association between techno-complexity and burnout in TEL may not be as strong for them as for the students in social sciences. However, this argument should not be interpreted with a derogatory connotation because each academic discipline has its own merits and we are not seeking to argue which way of training is superior.

### 5.1. Contributions

This study may contribute to current research and practice on technostress, which are often not discussed to an extant in institutional and scholastic efforts toward digitalized education, in the following ways. First, by conceptualizing problems (e.g., overload, physical/psychological strains, and disrupted routine practices) associated with the implementation of TEL in higher education in terms of the idea of technostress, this study provides a new perspective to understanding the imbalanced interplay between students and the implementation of TEL and therefore, suggests alternative solutions to the implementation problems that go beyond introducing newer and/or more advanced technology, for instance, setting clear expectations and requirements of TEL and aligning them with students’ capabilities.

Second, this study has demonstrated the suitability and effectiveness of the SSO model in unravelling how technostress may affect university students in TEL, thus contributing to the advancement of theories for investigating technostress in higher education. Third, this study contributes to the limited but emerging stream of research on possible downsides associated with the integration of technology in higher education. By examining how technostress creators in TEL impact university students’ learning through the SSO framework, the present study provides evidence-based arguments for more future scholastic and practical efforts to tackle this issue and also informs future implementation decisions related to TEL.

Finally, the group comparisons based on gender, academic disciplines, and willingness to join TEL contribute new insights into how the effects of technostress on university students’ learning in TEL may vary based on these factors, thereby enriching our knowledge of the functioning of technostress and subsequently, inspiring the development of targeted countermeasures against this issue. For instance, instead of mandating TEL for all students, universities may consider allowing students more choices regarding course solutions or more forms of TEL so as to appeal to their learning habits and preferences and decrease the chance of suffering technostress.

### 5.2. Implications

The current study carries the following implications for educators, administrators, and students in higher education. First, the findings of the negative associations between technostress and students’ learning in TEL underscore the importance of adopting a more practical and realistic, instead of idealistic and aspirational, expectations of and perspectives on the integration of technology in higher education for educational practitioners with an interest in using technology to transform learning and teaching.

Second, the technostress creators examined in this study are not related to technical issues in essence. Instead, they were mostly caused by insufficient skills of adapting to TEL or lack of pedagogical designs with regard to the integration of technology in the classroom. Thus, university commitments toward improving the digital literacy of students and educators need to address not only technical skills but also pedagogical skills in the use of technology for learning and teaching, in particular. In doing so, educators in higher education can design and implement TEL in a way that is more suitable to students’ needs and capabilities. As a result, students can manage to adapt to TEL in a healthier manner. The balance between TEL’s requirements and students’ capabilities will maximally eradicate the source of technostress [9].

Third, institutional efforts seeking successful implementation of TEL are advised to take into consideration the mediation factors such as gender, academic disciplines, and willingness to join TEL, instead of taking a one-size-fits-all approach. For instance, considering that male students and those who are compelled to join TEL are more likely to experience burnout caused by technostress, greater efforts are suggested to guide these students in adapting to TEL. Furthermore, universities are advised to allow students more flexibility and power to determine their engagement into TEL, instead of pushing the agenda of digitalized education mainly for the sake of institutional interests [2].

### 5.3. Limitations and Future Research

Nevertheless, the interpretation of the findings of this study should consider the following limitations. First, although the issue of technostress was contextualized in the implementation of TEL in this study, data were collected at a given point. Therefore, causal relationships among technostress creators and strain and psychological outcomes cannot be obtained. Longitudinal studies are suggested to further validate the findings of this study to achieve causal relationships. Second, cultural differences may exist among students from different universities and cultures in their perceptions of technostress and potential negative effects of technostress on students’ psychological and behavioral responses. More follow-up studies are needed to examine the generalizability of this study’s findings. Third, this study only included self-reported measures and did not consider actual learning outcomes or objectively observed learning-related behaviors. Future studies may consider validating the research findings by collecting these types of data. Fourth, although the technostress creators examined in this study are typical and have been reported in many previous studies, they are not exhaustive. As such, future research may consider exploring more types of technostress creators related to the use of technology in higher education so as to provide a more comprehensive understanding of how they affect university students’ learning. Fifth, informed but also constrained by the SSO model, this study did not fully consider all possible relationships among technostress creators, strain, and outcomes. Thus, future studies are suggested to further explore the relationships among these variables (and/or their variants) while considering alternative theories so as to gain a better understanding of technostress and its consequences for learners’ wellbeing and performance in TEL.

## 6. Conclusions

Consistent with the SSO model of technostress, when individuals cannot cope with the demands that TEL imposes on them, they may suffer from technostress, which could cause physical or psychological discomfort and exhaustion. As individuals have tendencies to avoid things that cause discomfort for them and behaviors that intensify their psychological strains, they may readjust their learning engagement and reduce their willingness to persist in TEL once they perceive strong psychological strains resulting from an incapability to deal with challenges of TEL in a healthy way. In this regard, due attention from educators and other practitioners in universities is needed to contemplate the use of technology for serious learning and readjust strategies of implementing TEL in a way that fits students’ actual needs and capabilities so as to support their effective learning and maintain their psychological/physical health.

## Figures and Tables

**Table 1:** Cronbach’s alpha, composite reliability, average variance extracted (AVE), and factor loadings of the SSO model.

Constructs/Items	Factor Loadings	M (SD)	Cronbach’s Alpha	Composite Reliability	AVE
Techno-overload			0.91	0.94	0.78
TO1	0.86	2.25 (0.89)			
TO2	0.88	2.22 (0.89)			
TO3	0.91	2.22 (0.90)			
TO4	0.90	2.21 (0.88)			
Techno-invasion			0.91	0.94	0.79
TIV1	0.90	2.24 (0.92)			
TIV2	0.89	2.24 (0.90)			
TIV3	0.89	2.19 (0.92)			
TIV4	0.88	2.15 (0.94)			
Techno-complexity			0.87	0.92	0.79
TC1	0.90	2.19 (0.89)			
TC2	0.88	2.26 (0.92)			
TC3	0.88	2.08 (0.94)			
Techno-insecurity			0.85	0.91	0.76
TIS1	0.90	2.15 (0.91)			
TIS2	0.85	2.27 (0.89)			
TIS3	0.87	2.15 (0.94)			
Techno-uncertainty			0.94	0.95	0.80
TU1	0.90	2.13 (0.93)			
TU2	0.89	2.12 (0.94)			
TU3	0.89	2.09 (0.92)			
TU4	0.91	2.11 (0.93)			
TU5	0.87	2.15 (0.93)			
Burnout in TEL			0.95	0.96	0.79
BN1	0.86	2.17 (0.89)			
BN2	0.90	2.09 (0.91)			
BN3	0.91	1.98 (0.93)			
BN4	0.88	2.11 (0.93)			
BN5	0.91	2.01 (0.93)			
BN6	0.88	2.03 (0.94)			
Self-regulation in TEL (reversed)			0.93	0.95	0.75
SR1	0.86	1.77 (0.84)			
SR2	0.87	1.81 (0.86)			
SR3	0.88	1.80 (0.86)			
SR4	0.84	1.72 (0.87)			
SR5	0.87	1.79 (0.85)			
SR6	0.86	1.73 (0.87)			
Learning agency in TEL (reversed)			0.92	0.95	0.82
LA1	0.91	1.66 (0.84)			
LA2	0.92	1.67 (0.85)			
LA3	0.90	1.64 (0.86)			
LA4	0.89	1.65 (0.83)			
Persistence in TEL (reversed)			0.93	0.95	0.78
PER1	0.88	1.62 (0.86)			
PER2	0.88	1.64 (0.87)			
PER3	0.89	1.64 (0.88)			
PER4	0.88	1.66 (0.86)			
PER5	0.89	1.64 (0.84)			

**Table 2:** Discriminant validity of the research model.

Constructs	1	2	3	4	5	6	7	8	9
1. Techno-overload	**0.88**								
2. Techno-invasion	0.84	**0.89**							
3. Techno-complexity	0.77	0.85	**0.89**						
4. Techno-insecurity	0.79	0.83	0.87	**0.87**					
5. Techno-uncertainty	0.69	0.74	0.82	0.84	**0.89**				
6. Burnout	0.53	0.58	0.65	0.64	0.65	**0.89**			
7. Self-regulation in TEL	−0.45	−0.45	−0.39	−0.43	−0.38	−0.46	**0.86**		
8. Learning agency in TEL	−0.43	−0.38	−0.33	−0.37	−0.33	−0.32	0.73	**0.90**	
9. Persistence in TEL	−0.41	−0.38	−0.33	−0.35	−0.28	−0.35	0.74	0.85	**0.88**

Note. The bold values in the diagonal row are the square roots of the average variance extracted for the constructs in the research model.

**Table 3:** Bootstrap validated outcomes of the SSO model.

Hypotheses		Path Coefficients	Standard Error	Percentile 0.025	Percentile 0.975	Results
H1	Techno-overload -> Burnout	−0.01 ns	0.06	−0.10	0.14	Not support
H2	Techno-invasion -> Burnout	0.02 ns	0.08	−0.15	0.14	Not support
H3	Techno-complexity -> Burnout	0.25 ***	0.05	0.14	0.34	Support
H4	Techno-insecurity -> Burnout	0.15 *	0.07	0.05	0.30	Support
H5	Techno-uncertainty -> Burnout	0.31 ***	0.06	0.18	0.40	Support
H6	Burnout -> Self-regulation	−0.46 ***	0.05	−0.57	−0.38	Support
H7	Burnout -> Learning agency	−0.32 ***	0.06	−0.44	−0.21	Support
H8	Burnout-> Persistence	−0.35 ***	0.06	−0.47	−0.24	Support

Note. * *p* < *0*.05; *** *p* < *0*.001; ns = nonsignificant; Persistence = Persistence in TEL.

**Table 4:** Comparison between male and female students.

Hypotheses		Global	Group: Females	Group: Males	*diff.abs*	*t*	*df*	*p*
H1	Techno-overload -> Burnout	−0.01	−0.02	−0.003	0.02	0.22	794	0.41
H2	Techno-invasion -> Burnout	0.02	−0.02	0.09	0.11	0.69	794	0.25
H3	Techno-complexity -> Burnout	0.25	0.30	0.18	0.12	0.86	794	0.20
H4	Techno-insecurity -> Burnout	0.15	0.15	0.14	0.01	0.02	794	0.49
H5	Techno-uncertainty -> Burnout	0.31	0.28	0.36	0.08	0.65	794	0.26
**H6**	**Burnout -> Self-regulation**	−0.46	**−0.31**	**−0.60**	**0.29**	**2.59**	**794**	**0.01**
**H7**	**Burnout -> Learning agency**	−0.32	**−0.18**	**−0.45**	**0.27**	**2.45**	**794**	**0.01**
**H8**	**Burnout-> Persistence**	−0.35	**−0.20**	**−0.48**	**0.28**	**2.50**	**794**	**0.01**

Note. *diff.abs* = absolute difference; the bold rows indicate the paths where male students significantly differed from female students.

**Table 5:** Comparison between students from social sciences (*N* = 334) and engineering and natural sciences (*N* = 462).

Hypotheses		Global	Group: SS	Group: EN	*diff.abs*	*t*	*df*	*p*
H1	Techno-overload -> Burnout	−0.01	0.07	−0.06	0.13	1.02	794	0.16
H2	Techno-invasion -> Burnout	0.02	−0.02	0.08	0.11	0.66	794	0.25
**H3**	**Techno-complexity -> Burnout**	**0.25**	**0.37**	**0.13**	**0.24**	**1.94**	**794**	**0.03**
H4	Techno-insecurity -> Burnout	0.15	0.12	0.19	0.07	0.55	794	0.29
H5	Techno-uncertainty -> Burnout	0.31	0.22	0.38	0.16	1.27	794	0.10
H6	Burnout -> Self-regulation	−0.46	−0.41	−0.50	0.09	0.91	794	0.18
H7	Burnout -> Learning agency	−0.32	−0.24	−0.36	0.12	1.13	794	0.13
H8	Burnout-> Persistence	−0.35	−0.25	−0.41	0.16	1.46	794	0.07

Note. SS = social sciences; EN = engineering and natural sciences; *diff.abs* = absolute difference; the bold rows indicate the paths where the students of social sciences significantly differed from those of engineering and natural sciences.

**Table 6:** Comparison between students who joined TEL willingly (*N* = 568) and unwillingly (*N* = 228).

Hypotheses		Global	Group: Willingly	Group: Unwillingly	*diff.abs*	*t*	*df*	*p*
H1 ^#^	Techno-overload -> Burnout	−0.01	0.18	−0.08	0.26	1.83	794	0.03
H2	Techno-invasion -> Burnout	0.02	0.07	0.02	0.05	0.27	794	0.39
H3	Techno-complexity -> Burnout	0.25	0.29	0.23	0.06	0.49	794	0.31
H4	Techno-insecurity -> Burnout	0.15	0.10	0.17	0.07	0.47	794	0.32
H5	Techno-uncertainty -> Burnout	0.31	0.19	0.33	0.14	0.96	794	0.17
**H6**	**Burnout -> Self-regulation**	**−0.46**	**−0.69**	**−0.36**	**0.33**	**2.94**	**794**	**0.00**
**H7**	**Burnout -> Learning agency**	**−0.32**	**−0.64**	**−0.18**	**0.46**	**3.75**	**794**	**0.00**
**H8**	**Burnout -> Persistence**	**−0.35**	**−0.67**	**−0.22**	**0.45**	**4.12**	**794**	**0.00**

Note. *diff.abs* = absolute difference; the bold rows indicate the paths where students who joined TEL willingly significantly differed from those who joined TEL unwillingly; # = As techno-overload did not significantly predict burnout in TEL (path coefficient of −0.03) for the whole sample, the two sub-datasets cannot be regarded as significantly different on the path relationship.

## Data Availability

The data presented in this study are available on request from the corresponding author. The data are not publicly available due to ethical reasons.
